# Supplementary material for: Landscape of the Epstein-Barr virus-host chromatin interactome and gene regulation
Source: EMBO J. 2025 May 27;44(13):3872–915. doi: 10.1038/s44318-025-00466-5 (PMC12216251; doi:10.1038/s44318-025-00466-5)
Supplement: Supplementary file 5 — Movie EV1 [file 44318_2025_466_MOESM5_ESM.zip › Movie EV1.docx]

**Movie EV1. Three-dimensional (3D) model of EBV.**

(Related to Fig. 1C). This model illustrates the higher-order organization of the EBV genome, featuring a platform-like structure with structural foci connected by chromatin loop anchors and prominent outward loops. Bead colors correspond to the linear genome localization shown in the color bar in Fig. 1B, with highlighted colors indicating specific regions of interest: magenta represents R2, green represents R1, black represents *EBERs*, and blue represents *miR-BARTs*.
