# Supplementary material for: Landscape of the Epstein-Barr virus-host chromatin interactome and gene regulation
Source: EMBO J. 2025 May 27;44(13):3872–915. doi: 10.1038/s44318-025-00466-5 (PMC12216251; doi:10.1038/s44318-025-00466-5)
Supplement: Supplementary file 6 — Movie EV2 [file 44318_2025_466_MOESM6_ESM.zip › Movie EV2.docx]

**Movie EV2. 3D BALM of EBV genome.**

(Related to Fig. 1E). The Movie displays the ring-shaped configuration of EBV genome. The structure was reconstructed using BALM. The color scale indicates the z-axis range, highlighting the spatial height distribution. The scale bar corresponds to 1000 nm.
