# Supplementary material for: Landscape of the Epstein-Barr virus-host chromatin interactome and gene regulation
Source: EMBO J. 2025 May 27;44(13):3872–915. doi: 10.1038/s44318-025-00466-5 (PMC12216251; doi:10.1038/s44318-025-00466-5)
Supplement: Supplementary file 7 — Movie EV3 [file 44318_2025_466_MOESM7_ESM.zip › Movie EV3.docx]

**Movie EV3. 3D BALM of EBV DNA.**

(Related to Fig. 1F). The Movie displays the linear configuration of purified EBV DNA. The structure was reconstructed using BALM. The color scale represents the z-axis range, emphasizing the distribution of height variations within the DNA structure. The scale bar corresponds to 1000 nm.
