# Supplementary figures and images for: Landscape of the Epstein-Barr virus-host chromatin interactome and gene regulation
Source: EMBO J. 2025 May 27;44(13):3872–915. doi: 10.1038/s44318-025-00466-5 (PMC12216251; doi:10.1038/s44318-025-00466-5)

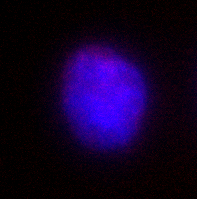

Supplement: Supplementary file 8 — Source data Fig. 1 [file 44318_2025_466_MOESM8_ESM.zip › Figure 1/1H/Fig1H-Merge.tif]

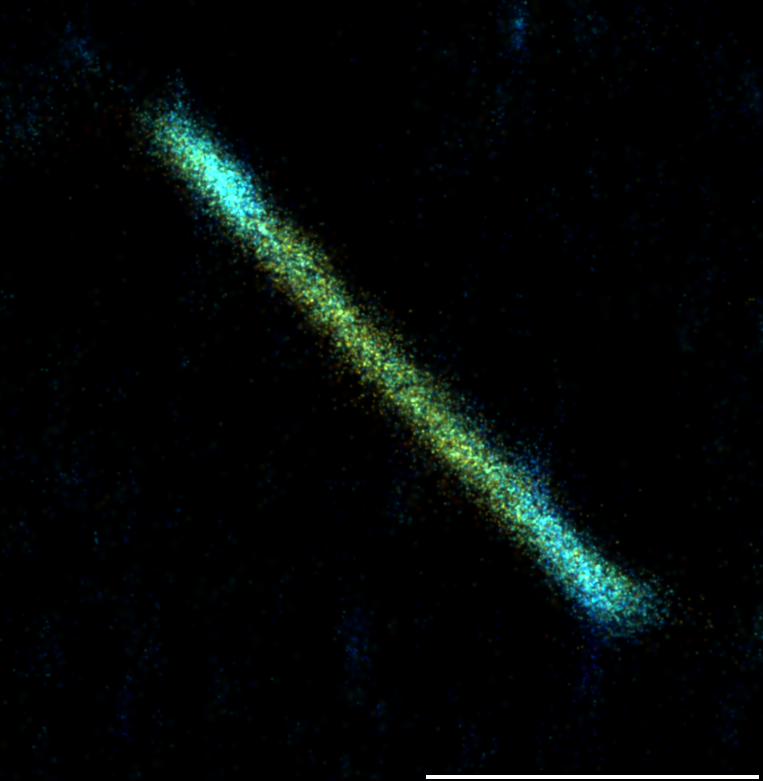

Supplement: Supplementary file 8 — Source data Fig. 1 [file 44318_2025_466_MOESM8_ESM.zip › Figure 1/1F/Fig1F.tif]

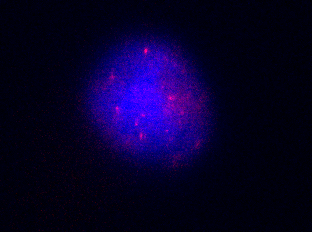

Supplement: Supplementary file 8 — Source data Fig. 1 [file 44318_2025_466_MOESM8_ESM.zip › Figure 1/1G/Fig1G-Merge.tif]

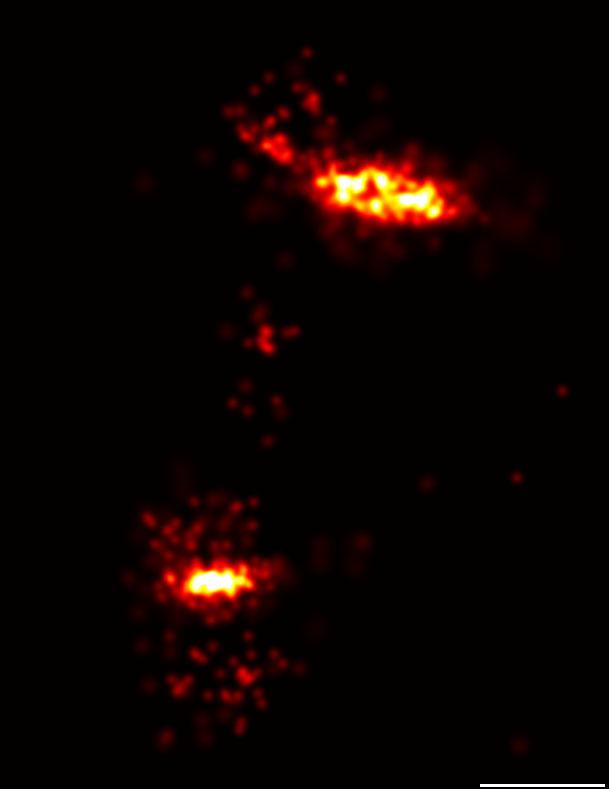

Supplement: Supplementary file 8 — Source data Fig. 1 [file 44318_2025_466_MOESM8_ESM.zip › Figure 1/1I/Fig1I.tif]

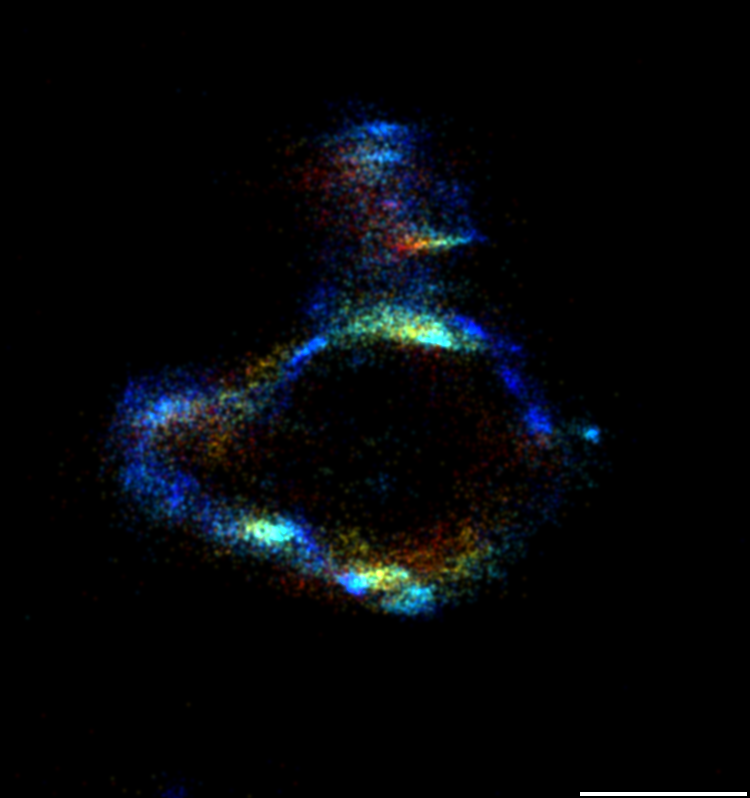

Supplement: Supplementary file 8 — Source data Fig. 1 [file 44318_2025_466_MOESM8_ESM.zip › Figure 1/1E/Fig1E.tif]

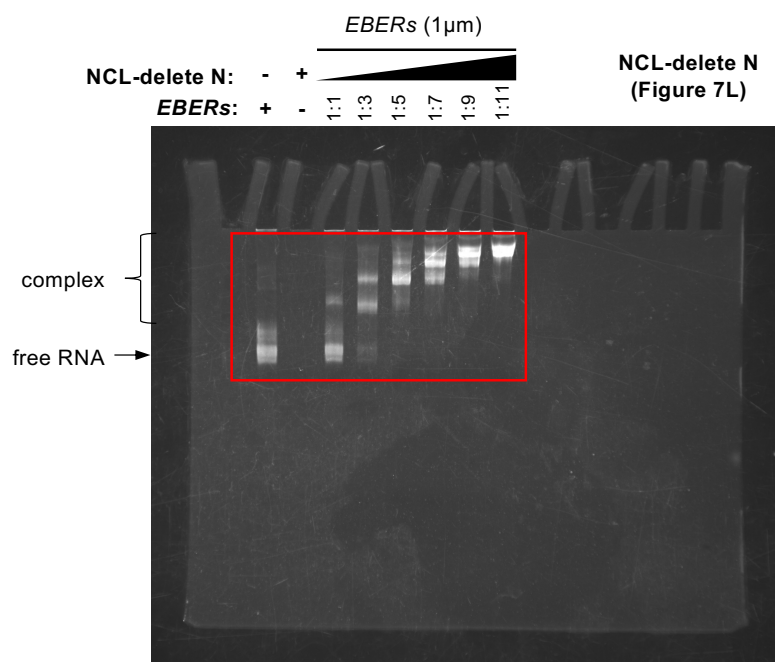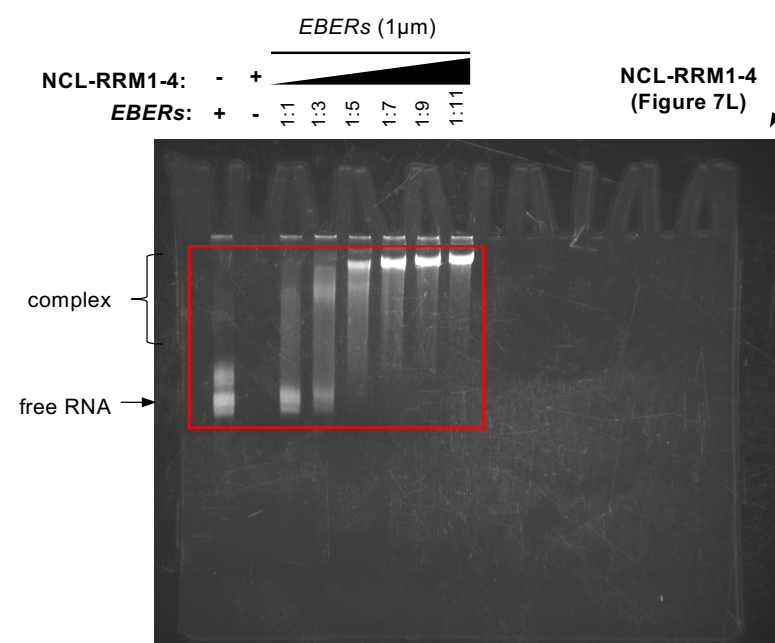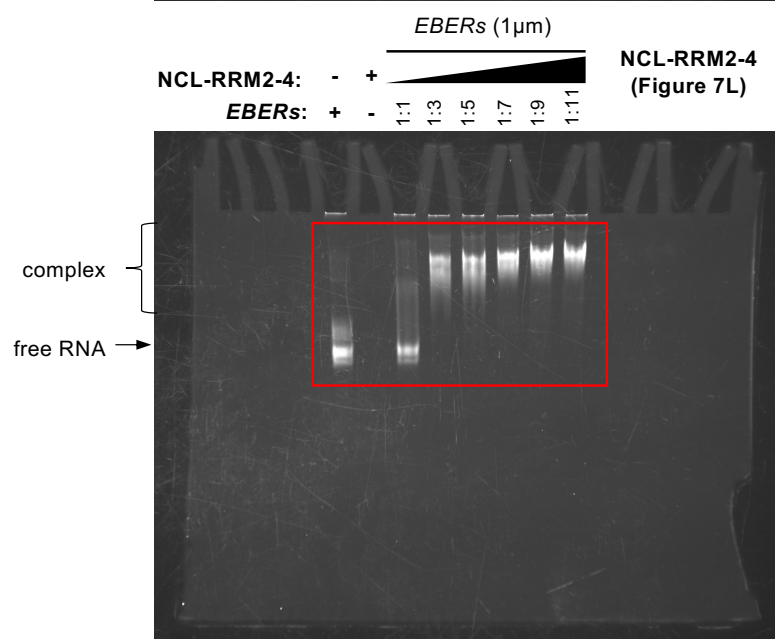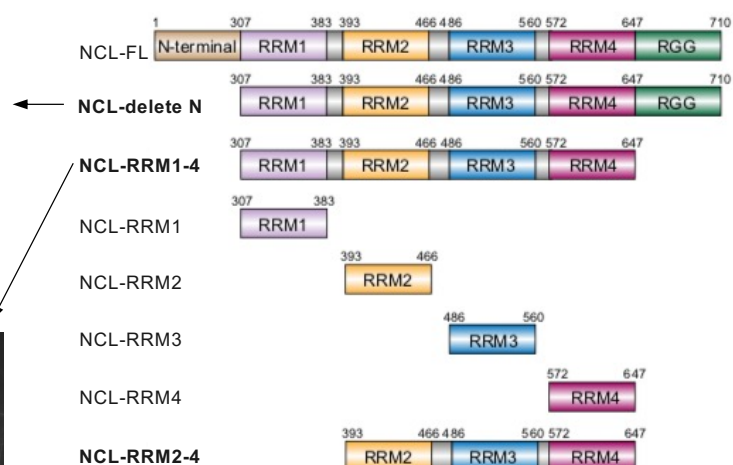

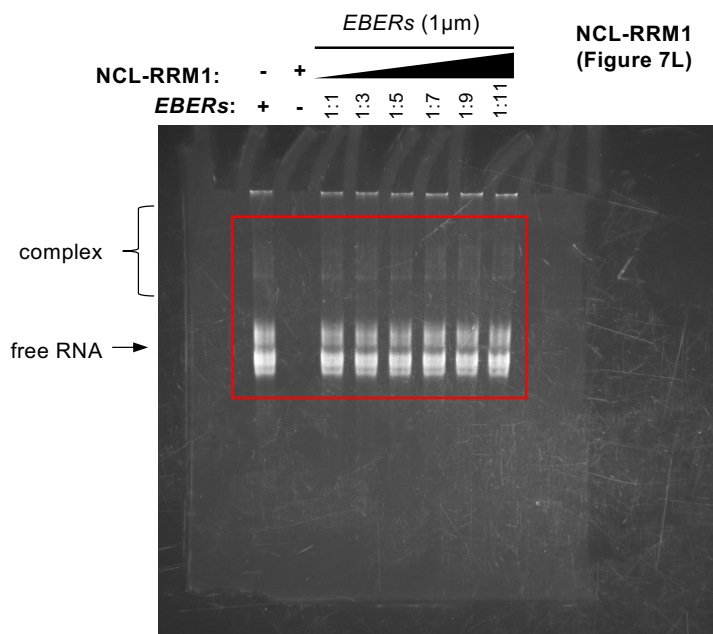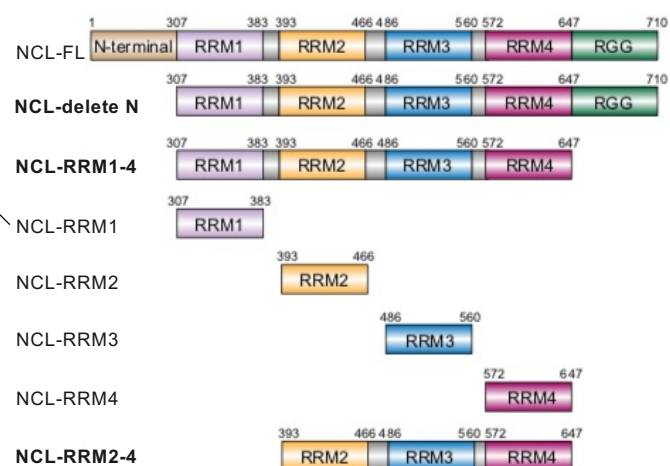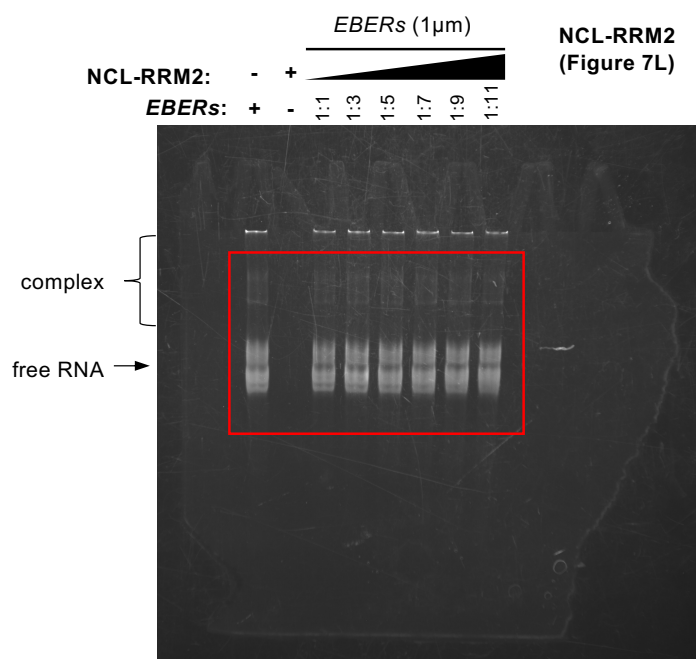

EBERs (1µm)

NCL-RRM3: - +

EBERs: + - 1:1 1:3 1:5 1:7 1:9 1:11

**NCL-RRM3**  
(Figure 7L)

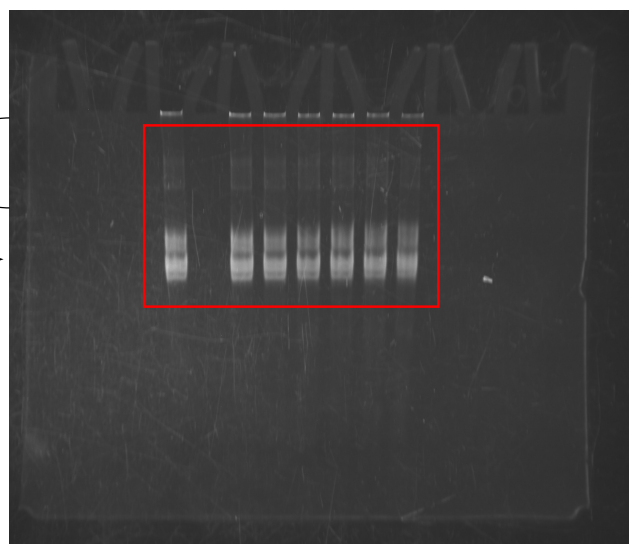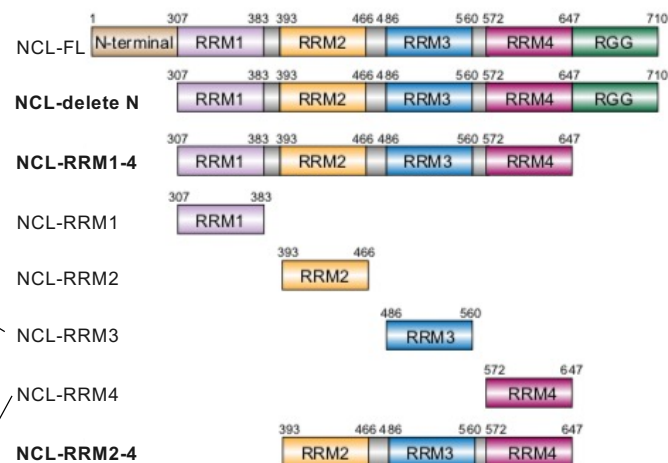

EBERs (1µm)

NCL-RRM4: - +

EBERs: + - 1:1 1:3 1:5 1:7 1:9 1:11

**NCL-RRM4**  
(Figure 7L)

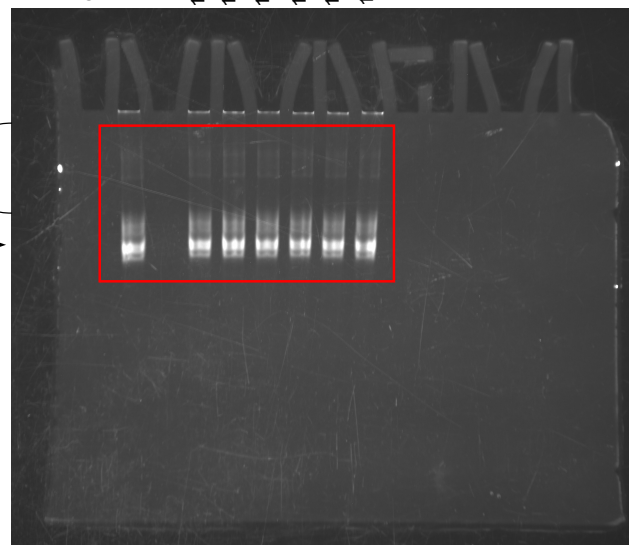

Supplement: Supplementary file 14 — Source data Fig. 7 [file 44318_2025_466_MOESM14_ESM.zip › Figure 7/7L/Fig7L.Gel_images.pdf]
