# Supplementary material for: Landscape of the Epstein-Barr virus-host chromatin interactome and gene regulation
Source: EMBO J. 2025 May 27;44(13):3872–915. doi: 10.1038/s44318-025-00466-5 (PMC12216251; doi:10.1038/s44318-025-00466-5)
Supplement: Supplementary file 16 — Expanded View Figures [file 44318_2025_466_MOESM16_ESM.pdf]

Expanded View Figures

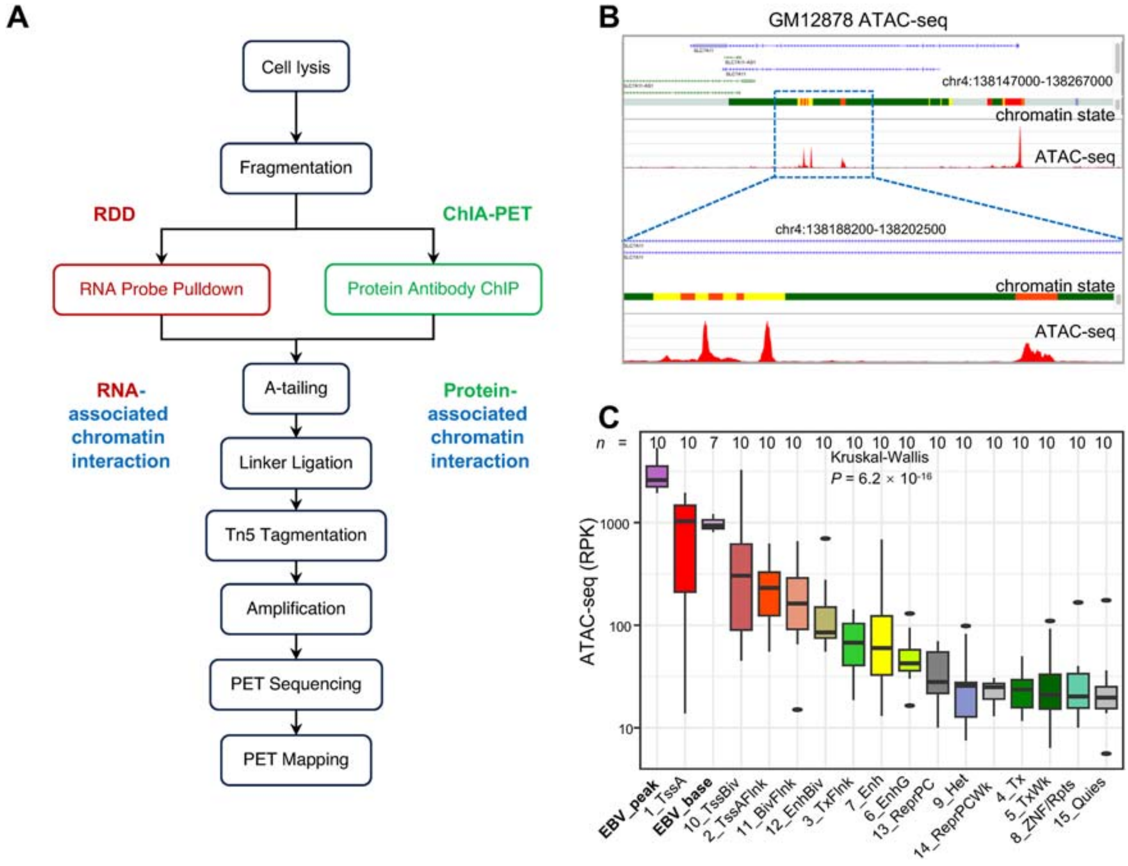

Figure EV1. Chromatin interactions and chromatin accessibility.

(**A**) Flowchart of chromatin interactions associated with protein factors detected by the ChIA-PET method and those associated with ncRNA factors from the RDD method. (**B**) BASIC Browser visualization of distinctive chromatin accessible and inaccessible regions in host human GM12878 cells from ATAC-seq data. (**C**) Box plots illustrating the chromatin accessibility across the 15 human chromatin states, as well as the EBV ATAC-seq peak region (EBV_peak) and non-peak region (EBV_base) indicating the baseline level. Chromatin state- 1: Active Transcription Start Site (TSS), 2: Flanking Active TSS, 3: Transcription at gene 5′ and 3′ ends, 4: Strong transcription, 5: Weak transcription, 6: Genic enhancers, 7: Enhancers, 8: ZNF genes and repeats, 9: Heterochromatin, 10: Bivalent/Poised TSS, 11: Flanking Bivalent TSS/Enhancers, 12: Bivalent Enhancer, 13: Repressed PolyComb, 14: Weak Repressed PolyComb, 15: Quiescent/Low. Kruskal–Wallis tests are performed, $P = 6.2 \times 10^{-14}$. Each box plot highlights the median (inside line), the 25-75th percentiles (box), and the minima/maxima values within 1.5× the interquartile range (IQR) of the box (whiskers).

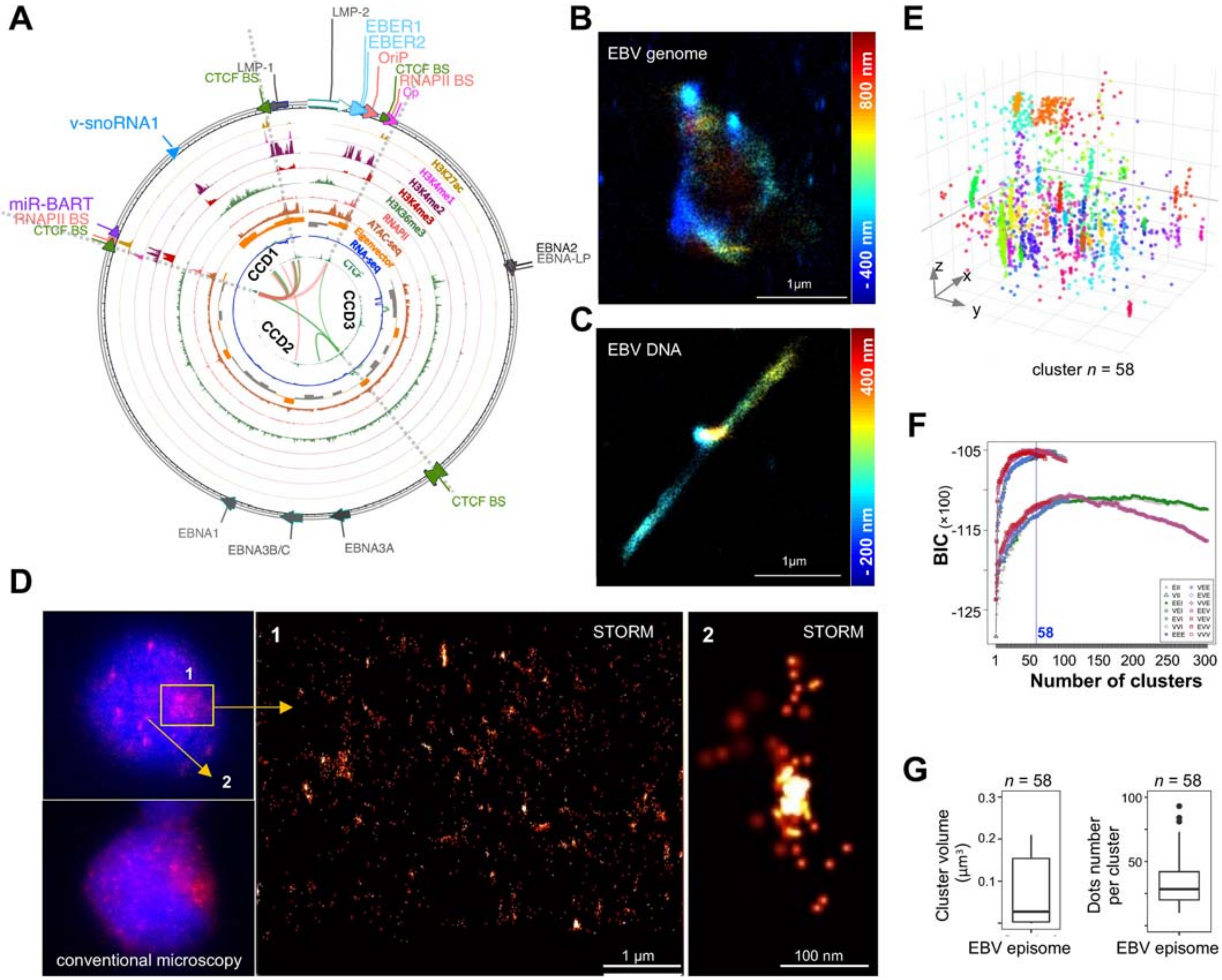

Figure EV2. EBV 3D structure.

(**A**) Circos plot displays CTCF- and RNAPII-mediated EBV chromatin interactions (in the innermost layer, with RNAPII-mediated loops in red and CTCF-mediated loops in green), along with histone marker signals. The outermost layer represents the EBV genome, with arrows indicating gene directions, and "CTCF BS" denoting CTCF-binding sites along with motif orientation. (**B**) Example images of BALM on EBV genome from B95-8 cells. The color scale corresponds to the *z* range. (**C**) Example images of BALM on EBV DNA. (**D**) Example images of conventional microscopy from DNA-FISH (red) of EBV and DAPI (blue) performed in GM12878 cells, along with STORM images showing a zoomed-in view of the EBV cloud highlighted by a yellow square labeled as '1' in the middle and EBV foci labeled as '2' on the right. Here, the STORM images presented the high-resolution structure from different loci of cells shown in Fig. 1G. (**E**) 3D structure of EBV clusters in the zoomed-in square in (**D**, middle), with each cluster corresponding to an EBV episome represented by a different color. (**F**) Bayesian information criterion (BIC) prediction method in the GMM (Gaussian mixture models) clustering algorithm for EBV clustering. (**G**) Box plots showing the density of EBV episome sizes (left panel) and the number of dots in individual EBV clusters (episomes, right panel). Each box plot highlights the median (inside line), the 25–75th percentiles (box), and the minima/maxima values within 1.5× the interquartile range (IQR) of the box (whiskers).

 © The Author(s)

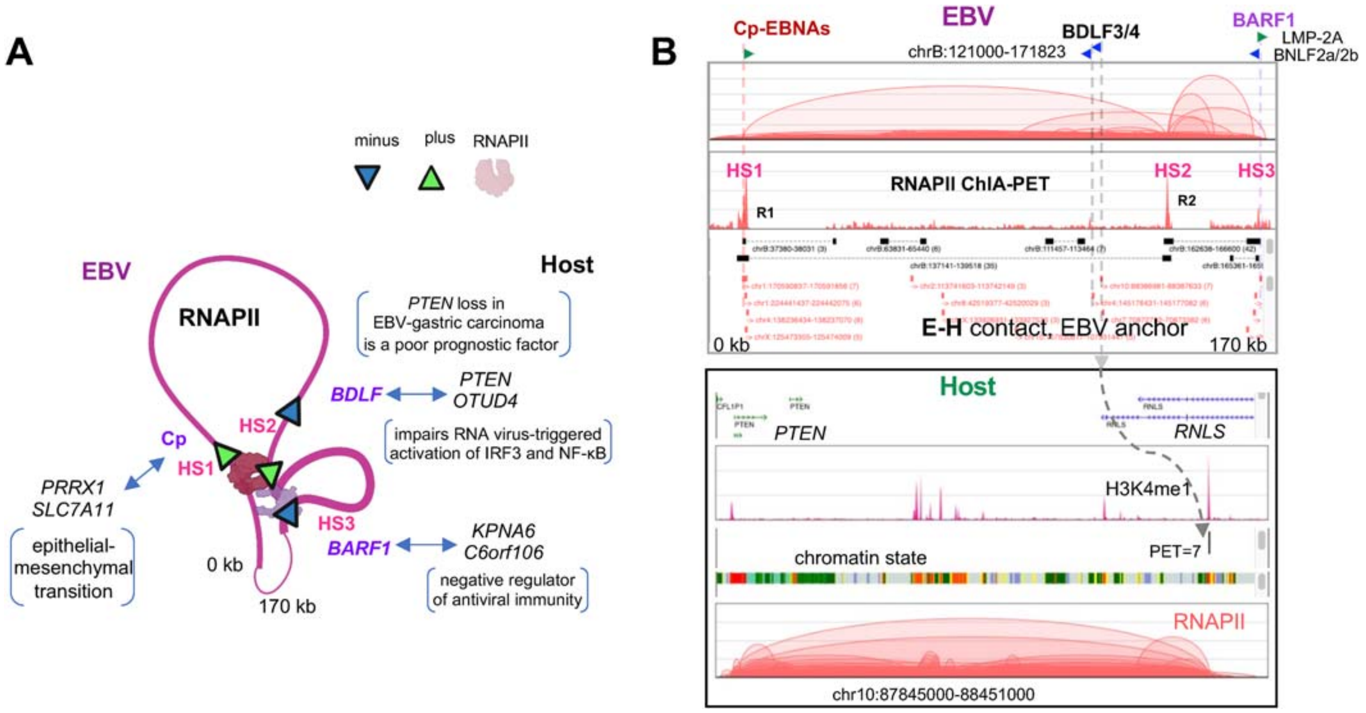

Figure EV3. Protein-associated E–H interaction.

(**A**) Diagram shows RNAPII-mediated chromatin interaction on EBV side. The arrow line represents the contact genes between EBV and the host; the parentheses indicate the function of the gene. Arrowhead presents gene transcribed orientation. (**B**) RNAPII-mediated chromatin interactions between EBV chromatin and host chromatin (E–H). EBV genes were highlighted on the top, followed by tracks for RNAPII loop, binding peak, and EBV interacting anchor. The dashed line points to the contact region of the host around *PTEN* gene, with H3K4me1 peak denoting the active enhancer region, alongside chromatin state and RNAPII-mediated loops.

© The Author(s)

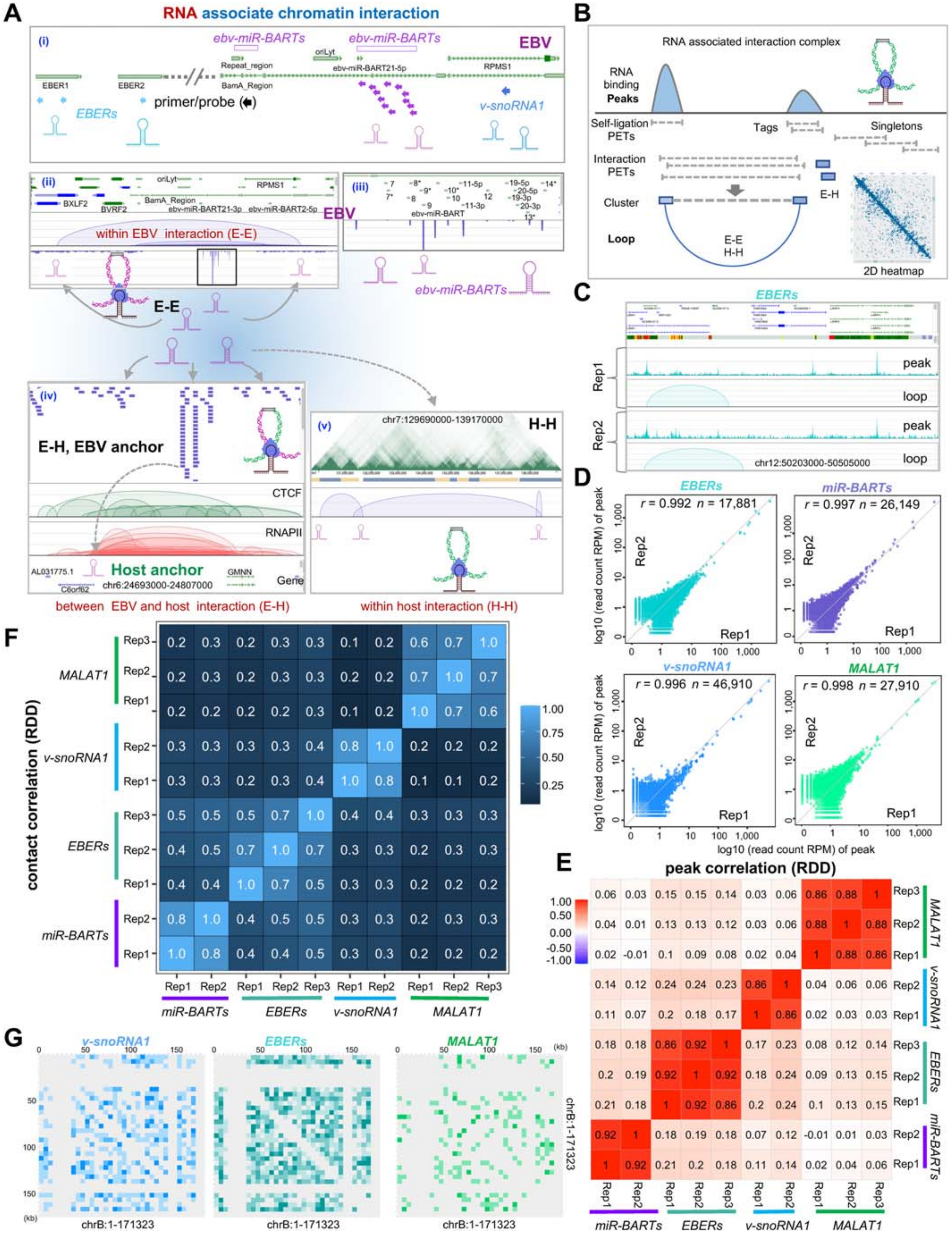
A
RNA associate chromatin interaction
(i)
ebv-miR-BARTs
ebv-miR-BARTs
EBV
oriLyt
Repeat_region
ebv-miR-BART21-5p
RPMS1
EBER1
EBER2
BamA_Region
primer/probe (⬅)
EBERs
v-snoRNA1
(ii)
oriLyt
RPMS1
BXLF2
BVRF2
BamA_Region
ebv-miR-BART21-3p
ebv-miR-BART2-5p
EBV
within EBV interaction (E-E)
(iii)
ebv-miR-BART
ebv-miR-BARTs
E-E
(iv)
E-H, EBV anchor
CTCF
RNAPII
AL031775.1
Host anchor
GMNN
C6orf62
chr6:24693000-24807000
Gene
(v)
chr7:129690000-139170000
H-H
between EBV and host interaction (E-H)
within host interaction (H-H)
B
RNA associated interaction complex
RNA binding Peaks
Self-ligation PETs
Tags
Singletons
Interaction PETs
E-H
Cluster
Loop
E-E
H-H
2D heatmap
C
EBERs
Rep1
peak
loop
Rep2
peak
chr12:50203000-50505000
loop
D
EBERs
r = 0.992 n = 17,881
miR-BARTs
r = 0.997 n = 26,149
v-snoRNA1
r = 0.996 n = 46,910
MALAT1
r = 0.998 n = 27,910
Rep2
Rep1
log10 (read count RPM) of peak
E
peak correlation (RDD)
MALAT1 Rep3: 0.06 0.03 0.15 0.15 0.14 0.03 0.06 0.86 0.88 1
MALAT1 Rep2: 0.04 0.01 0.13 0.13 0.12 0.03 0.06 0.88 1 0.88
MALAT1 Rep1: 0.02 -0.01 0.1 0.09 0.08 0.02 0.04 1 0.88 0.86
v-snoRNA1 Rep2: 0.14 0.12 0.24 0.24 0.23 0.86 1 0.04 0.06 0.06
v-snoRNA1 Rep1: 0.11 0.07 0.2 0.18 0.17 1 0.86 0.02 0.03 0.03
EBERs Rep3: 0.18 0.18 0.86 0.92 1 0.17 0.23 0.08 0.12 0.14
EBERs Rep2: 0.2 0.19 0.92 1 0.92 0.18 0.24 0.09 0.13 0.15
EBERs Rep1: 0.21 0.18 1 0.92 0.86 0.2 0.24 0.1 0.13 0.15
miR-BARTs Rep2: 0.92 1 0.18 0.19 0.18 0.07 0.12 -0.01 0.01 0.03
miR-BARTs Rep1: 1 0.92 0.21 0.2 0.18 0.11 0.14 0.02 0.04 0.06
Rep1 Rep2 Rep1 Rep2 Rep3 Rep1 Rep2 Rep1 Rep2 Rep3
miR-BARTs EBERs v-snoRNA1 MALAT1
1.00 0.50 0.00 -0.50 -1.00
F
contact correlation (RDD)
MALAT1 Rep3: 0.2 0.3 0.2 0.3 0.3 0.1 0.2 0.6 0.7 1.0
MALAT1 Rep2: 0.2 0.3 0.2 0.3 0.3 0.2 0.2 0.7 1.0 0.7
MALAT1 Rep1: 0.2 0.2 0.2 0.2 0.3 0.1 0.2 1.0 0.7 0.6
v-snoRNA1 Rep2: 0.3 0.3 0.3 0.3 0.4 0.8 1.0 0.2 0.2 0.2
v-snoRNA1 Rep1: 0.3 0.3 0.2 0.3 0.4 1.0 0.8 0.1 0.1 0.2
EBERs Rep3: 0.5 0.5 0.5 0.7 1.0 0.4 0.4 0.3 0.3 0.3
EBERs Rep2: 0.4 0.5 0.7 1.0 0.7 0.3 0.3 0.2 0.3 0.3
EBERs Rep1: 0.4 0.4 1.0 0.7 0.5 0.3 0.3 0.2 0.2 0.2
miR-BARTs Rep2: 0.8 1.0 0.4 0.5 0.5 0.3 0.3 0.2 0.3 0.3
miR-BARTs Rep1: 1.0 0.8 0.4 0.4 0.5 0.3 0.3 0.2 0.2 0.2
Rep1 Rep2 Rep1 Rep2 Rep3 Rep1 Rep2 Rep1 Rep2 Rep3
miR-BARTs EBERs v-snoRNA1 MALAT1
1.00 0.75 0.50 0.25
G
v-snoRNA1
EBERs
MALAT1
(kb)
chrB:1-171323

 © The Author(s)

Figure EV4. The RDD method detects EBV ncRNA-associated chromatin interactions.

(**A**) Positions of primers/probes of ncRNAs along EBV genome that are used in this study (**i**), Arrowheads indicate the primer/probes direction, Hairpin RNA indicates transcripts of target RNAs (Cyan represents *EBERs*, Purple represents *miR-BARTs*, Light blue represents *v-snoRNA1*); (**ii**) Viral RNA-associated EBV chromatin DNA–DNA interaction loops along with EBV genome reference (EBV-to-EBV: E–E), square highlights target RNA origin transcript loci that were zoomed in (**iii**); (**iv**) Viral RNA facilitates the chromatin DNA–DNA interactions between EBV and host (E–H). Purple bar represents the contact anchor at EBV chromatin, dash line with arrowhead directs to the other contact anchor at host chromatin annotated with CTCF (green) and RNAPII (red) mediated chromatin loops. (**v**) Viral RNA-associated host chromatin DNA–DNA interaction loops (host-to-host: H–H) along with 2D heatmap contacts. (**B**) Graphic of RDD mapping properties including binding peaks piled up from all tags, self-ligation PETs (two ends of tags are from the same DNA fragment), interaction PETs (two ends of tags are from different DNA fragments), singletons (individual PETs), clusters or loops (overlapped PETs), interacting anchor between E–H or inter-chromosomes, and visualization of pairwise contacts in form of loops or contact heatmaps. (**C**) BASIC Browser visualization of EBV ncRNA *EBERs*-associated chromatin loops and peaks in two RDD replicates. (**D**–**F**) Reproducibility analyses of RDD data were conducted using different methods: (**D**) Scatter plots of RDD peaks between replicates. The *r* value represents the Pearson correlation coefficient. (**E**) the Spearman correlation coefficient of RDD peaks among all replicates, and (**F**) RDD contact data using HiCRep. The stratum-adjusted correlation coefficient (SCC) was computed for pairs of RDD libraries, with the SCC value displayed. “Rep” denotes replicate. (**G**) 2D heatmaps represent the EBV ncRNA-associated chromatin interactions within the EBV genome (E–E).

© The Author(s)

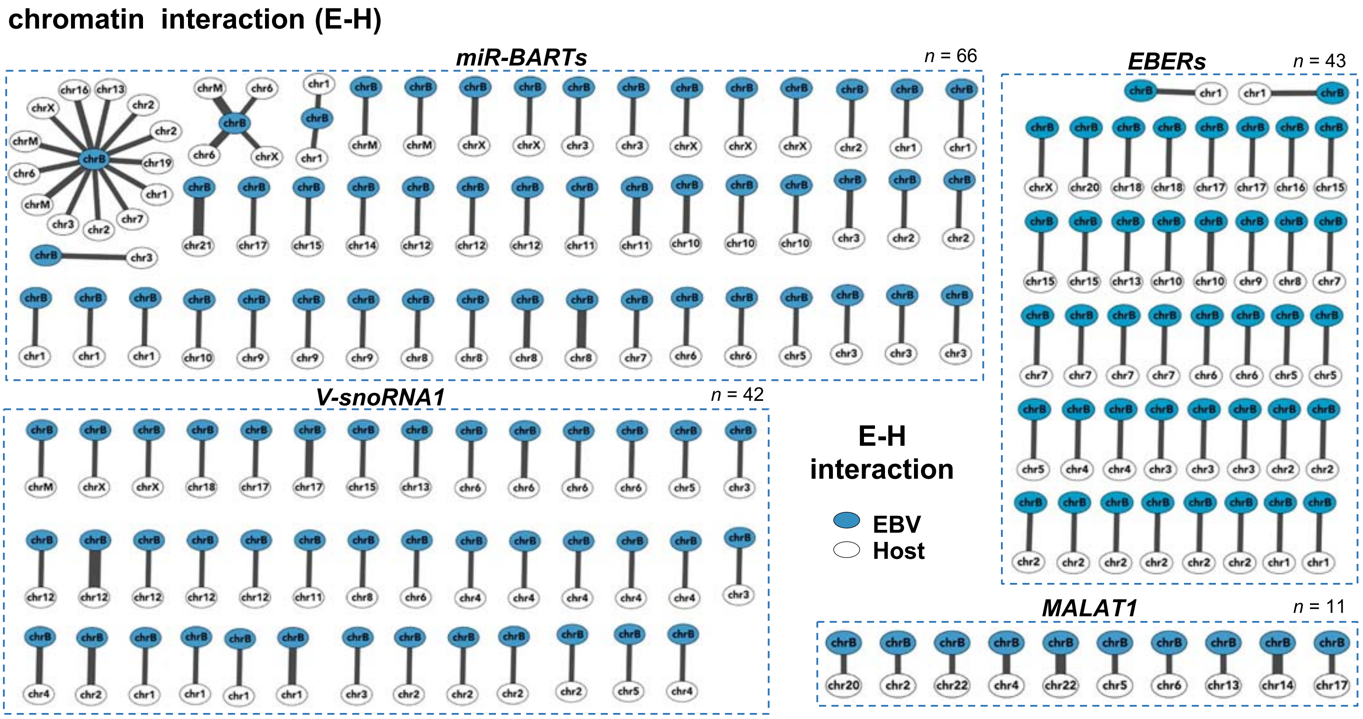

Figure EV5. EBV and host chromatin interaction network (E–H).

Cytoscape illustration of the contact network (PET ≥ 2) between EBV and the host (E–H) associated with ncRNA factors (*EBERs*, *miR-BARTs*, *v-snoRNA1*, and *MALAT1*). Blue ellipses represent EBV, labeled as “chrB,” while white ellipses represent the host, labeled as “chr#.” Most interactions involve a one-to-one correspondence between a “chrB” and a “chr#,” except for *miR-BARTs*, which show a single contact where one “chrB” corresponds to multiple “chr#“ instances. This indicates that the EBV episome primarily contacts the host chromatin in a one-to-one manner.

 © The Author(s)

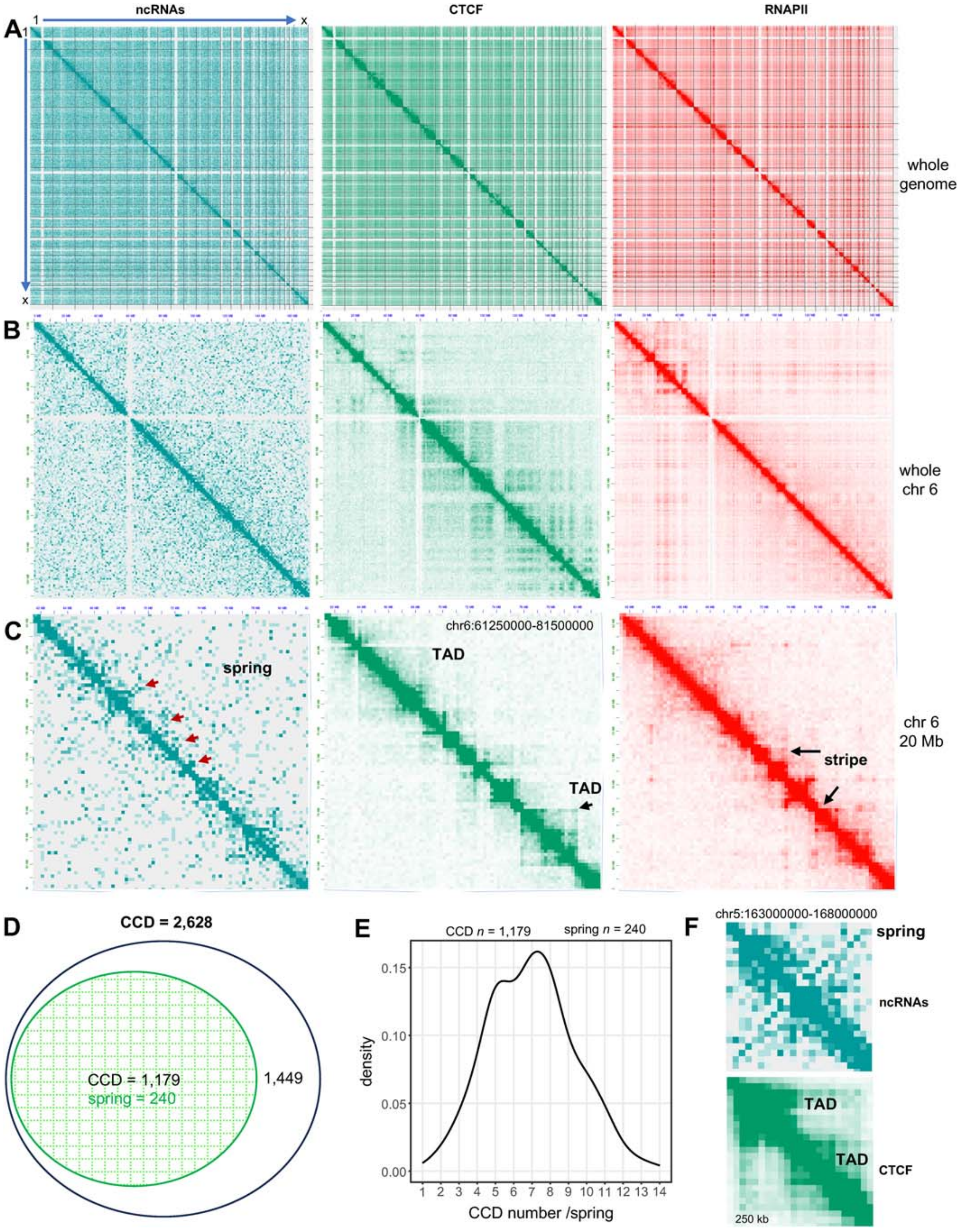
A
B
C
D
E
F
ncRNAs
CTCF
RNAPII
whole genome
whole chr 6
chr 6 20 Mb
chr6:61250000-81500000
spring
TAD
stripe
CCD = 2,628
CCD = 1,179
spring = 240
1,449
CCD n = 1,179
spring n = 240
density
CCD number /spring
chr5:163000000-168000000
250 kb

Figure EV6. Contact heatmaps present chromatin contact profiling associated with ncRNAs or proteins.

(**A–C**) Contact heatmaps cover the whole genome (**A**), whole chromosome level (**B**), and a zoomed-in genomic region spanning 20 Mb at 100 kb resolution (**C**), with chromatin structures of 'spring', 'TAD' and 'stripe' marked. (**D**) Venn diagram illustrates the number of CCDs covered by the springs. (**E**) Density plot displays the distribution of CCD numbers within individual springs. (**F**) Representative examples highlighting the relationship between springs and CCDs/TADs.

© The Author(s)

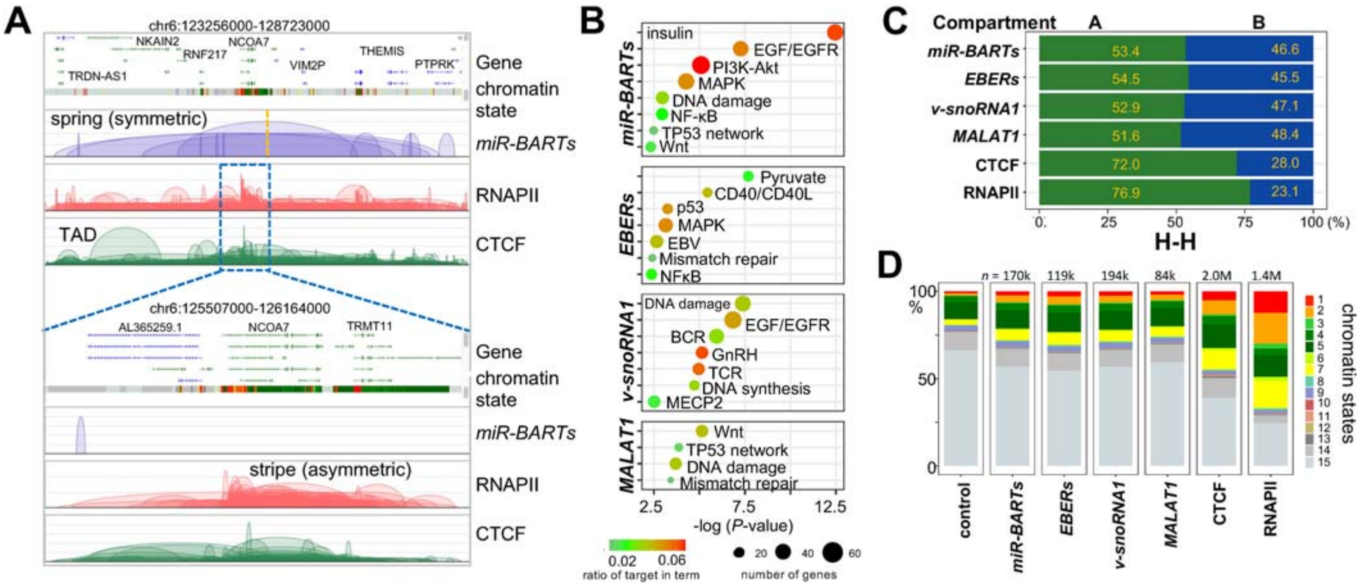

Figure EV7. EBV ncRNAs-associated chromatin interaction within host genome (H–H).

(**A**) BASIC Browser visualization of chromatin loop profiling involving ncRNA *BARTs*-associated and RNAPII/CTCF-mediated interactions. Yellow dashed lines highlight the ncRNA-associated chromatin symmetric midpoint, and dashed boxes provide a zoomed-in view of the RNAPII/CTCF-mediated chromatin loop structure. (**B**) GO term enrichment analysis of host chromatin loop-anchored genes targeted by ncRNAs. (**C**) Histogram shows percentage of chromatin loop-anchored targets in the A/B compartment. (**D**) Histogram shows the distribution of 15 chromatin states (see "Methods") for ncRNA-targeted host chromatin loops (H–H).

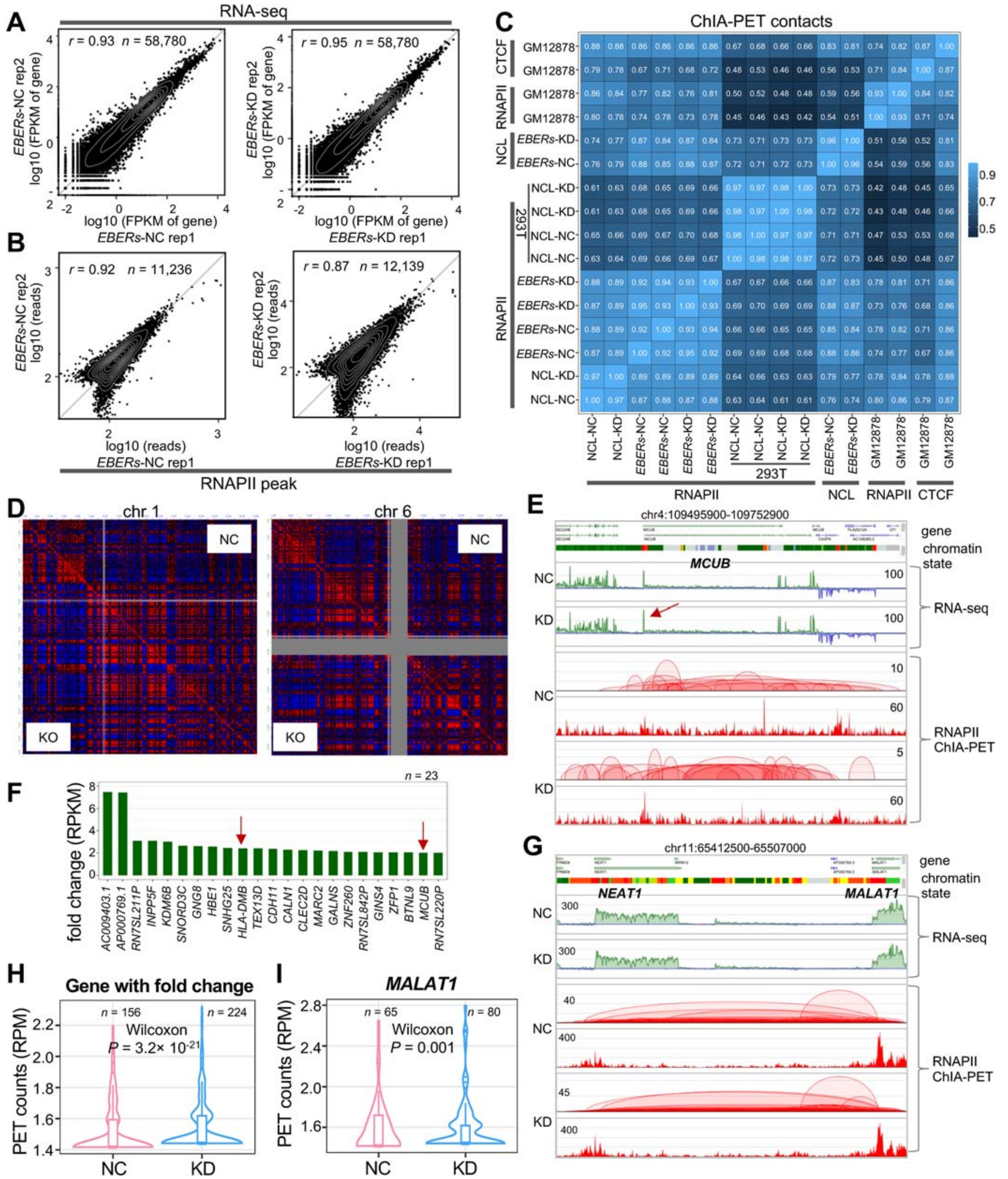
A
RNA-seq
r = 0.93 n = 58,780
EBERs-NC rep2 log10 (FPKM of gene)
log10 (FPKM of gene) EBERs-NC rep1
r = 0.95 n = 58,780
EBERs-KD rep2 log10 (FPKM of gene)
log10 (FPKM of gene) EBERs-KD rep1
B
r = 0.92 n = 11,236
EBERs-NC rep2 log10 (reads)
log10 (reads) EBERs-NC rep1
r = 0.87 n = 12,139
EBERs-KD rep2 log10 (reads)
log10 (reads) EBERs-KD rep1
RNAPII peak
C
ChIA-PET contacts
CTCF
RNAPII
NCL
293T
GM12878
EBERs-KD
EBERs-NC
NCL-KD
NCL-NC
NCL RNAPII CTCF
D
chr 1
chr 6
NC
KO
E
chr4:109495900-109752900
MCUB
gene
chromatin state
RNA-seq
RNAPII ChIA-PET
F
n = 23
fold change (RPKM)
AC009403.1 AP000769.1 RN7SL211P INPP5F KDM6B SNORD3C GNG8 HBE1 SNHG25 HLA-DMB TEX13D CDH11 CALN1 CLEC2D MARC2 GALNS ZNF260 RN7SL842P GINS4 ZFP1 BTNL9 MCUB RN7SL220P
G
chr11:65412500-65507000
NEAT1
MALAT1
H
Gene with fold change
n = 156
n = 224
Wilcoxon
P = 3.2× 10-21
PET counts (RPM)
I
MALAT1
n = 65
n = 80
Wilcoxon
P = 0.001
PET counts (RPM)
NC
KD

Figure EV8. EBV ncRNA *EBERs* repress host gene expression through RNAPII-associated chromatin loops.

(**A**, **B**) Reproducibility analyses of RNA-seq data (**A**) and RNAPII peak data (**B**) in *EBERs*-NC and *EBERs*-KD. The *r* value represents the Pearson correlation coefficient. "Rep" denotes replicate. (**C**) Reproducibility analyses of ChIA-PET contact data using HiCRep. The stratum-adjusted correlation coefficient (SCC) was computed for pairs of RDD libraries, with the SCC value displayed. (**D**) Observed Pearson Heatmaps for chromosome 1 and 6. The correlation matrix depicts the strength of correlation [from −1 (blue) to +1 (red)] between the intrachromosomal interaction profiles of every pair of 250-kb loci spanning the entire chromosome. The distinctive plaid pattern indicates the presence of A/B compartments within the chromosome. (**E**) Display of RNA expression levels and RNAPII-associated chromatin loops around the *MCUB* gene in *EBERs*-NC cells and *EBERs*-KD cells. (**F**) Bars presents the genes with more than a two-fold change expression when comparing *EBERs*-KD cells to *EBERs*-NC cells. Arrows indicate the *MCUB* and *HLA-DMB* genes, visualized with the BASIC Browser in (**E**) and Fig. 6D, respectively. (**G**) Display of RNA expression levels and RNAPII-associated chromatin loops around the *MALAT1* and *NEAT1* gene in *EBERs*-NC cells and *EBERs*-KD cells. (**H**) Violin-Box plots present the chromatin interaction PET counts from RNAPII ChIA-PET associated with the differentiated 231 genes in *EBERs*-KD cells versus *EBERs*-NC cells. Two-sided Wilcoxon tests are performed, $P = 3.2 \times 10^{-21}$. (**I**) Violin-Box plots present the chromatin interaction PET counts from RNAPII ChIA-PET in *EBERs*-NC cells and *EBERs*-KD cells at the genomic region of the *MALAT1* gene (**G**). Two-sided Wilcoxon tests are performed, $P = 0.001$. Each box plot highlights the median (inside line), the 25–75th percentiles (box), and the minima/maxima values within 1.5× the interquartile range (IQR) of the box (whiskers).

© The Author(s)

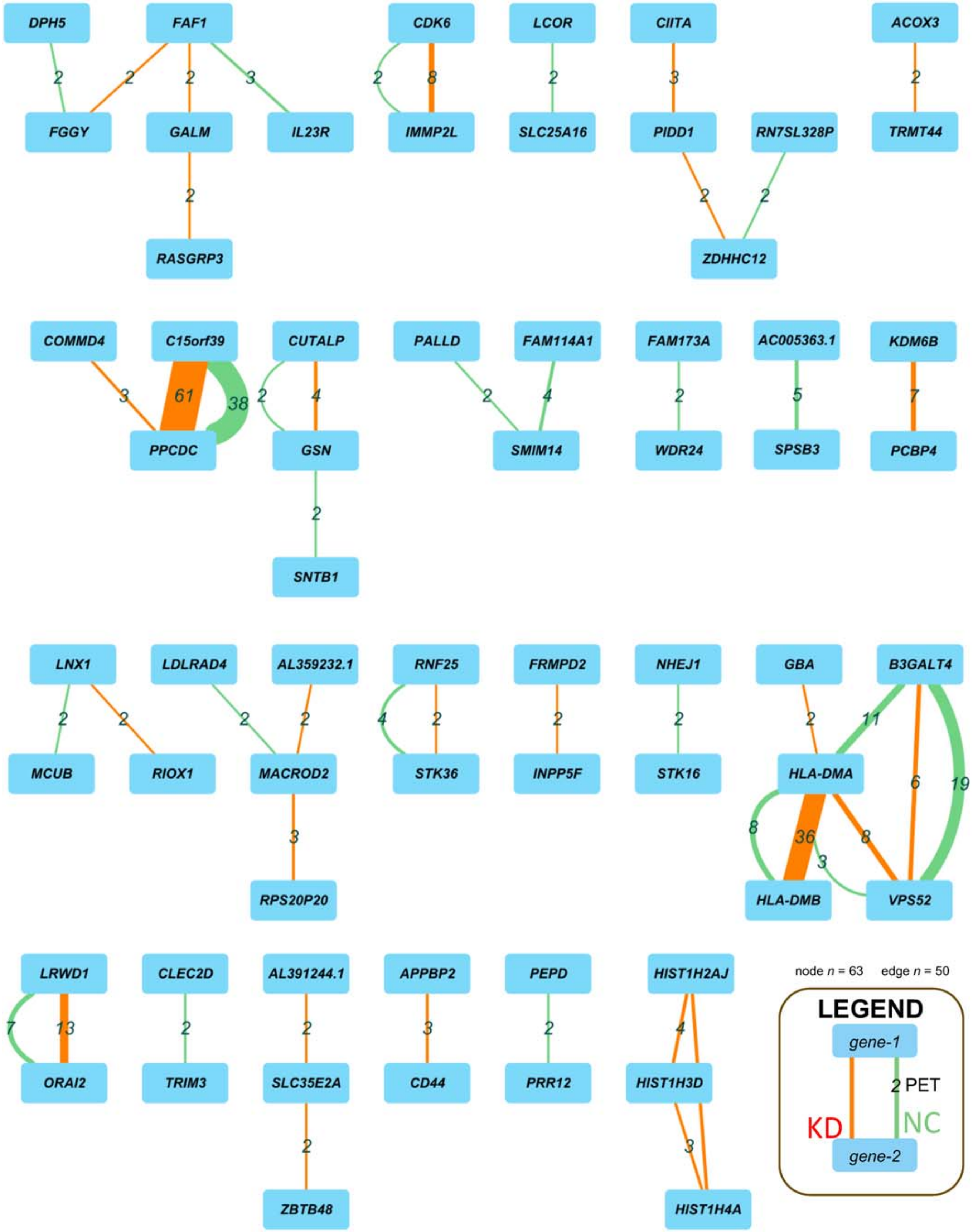
DPH5
FAF1
CDK6
LCOR
CIITA
ACOX3
FGGY
GALM
IL23R
IMMP2L
SLC25A16
PIDD1
RN7SL328P
TRMT44
RASGRP3
ZDHHC12
COMMD4
C15orf39
CUTALP
PALLD
FAM114A1
FAM173A
AC005363.1
KDM6B
PPCDC
GSN
SMIM14
WDR24
SPSB3
PCBP4
SNTB1
LNX1
LDLRAD4
AL359232.1
RNF25
FRMPD2
NHEJ1
GBA
B3GALT4
MCUB
RIOX1
MACROD2
STK36
INPP5F
STK16
HLA-DMA
RPS20P20
HLA-DMB
VPS52
LRWD1
CLEC2D
AL391244.1
APPBP2
PEPD
HIST1H2AJ
ORAI2
TRIM3
SLC35E2A
CD44
PRR12
HIST1H3D
ZBTB48
HIST1H4A
node n = 63 edge n = 50
LEGEND
gene-1
2 PET
KD
NC
gene-2

Figure EV9. The connections among genes repressed by *EBERs*.

Cytoscape illustrates the interaction network among the 231 *EBERs*-targeted genes captured by RNAPII ChIA-PET in *EBERs*-NC and *EBERs*-KD cells. The thickness of the edge reflects the interaction frequency. Orange color indicates RNAPII interaction loops in the *EBERs*-KD cells, while green color indicates RNAPII interaction loops in the *EBERs*-NC cells. The numbers represent PET counts, indicating the interaction frequency.

© The Author(s)

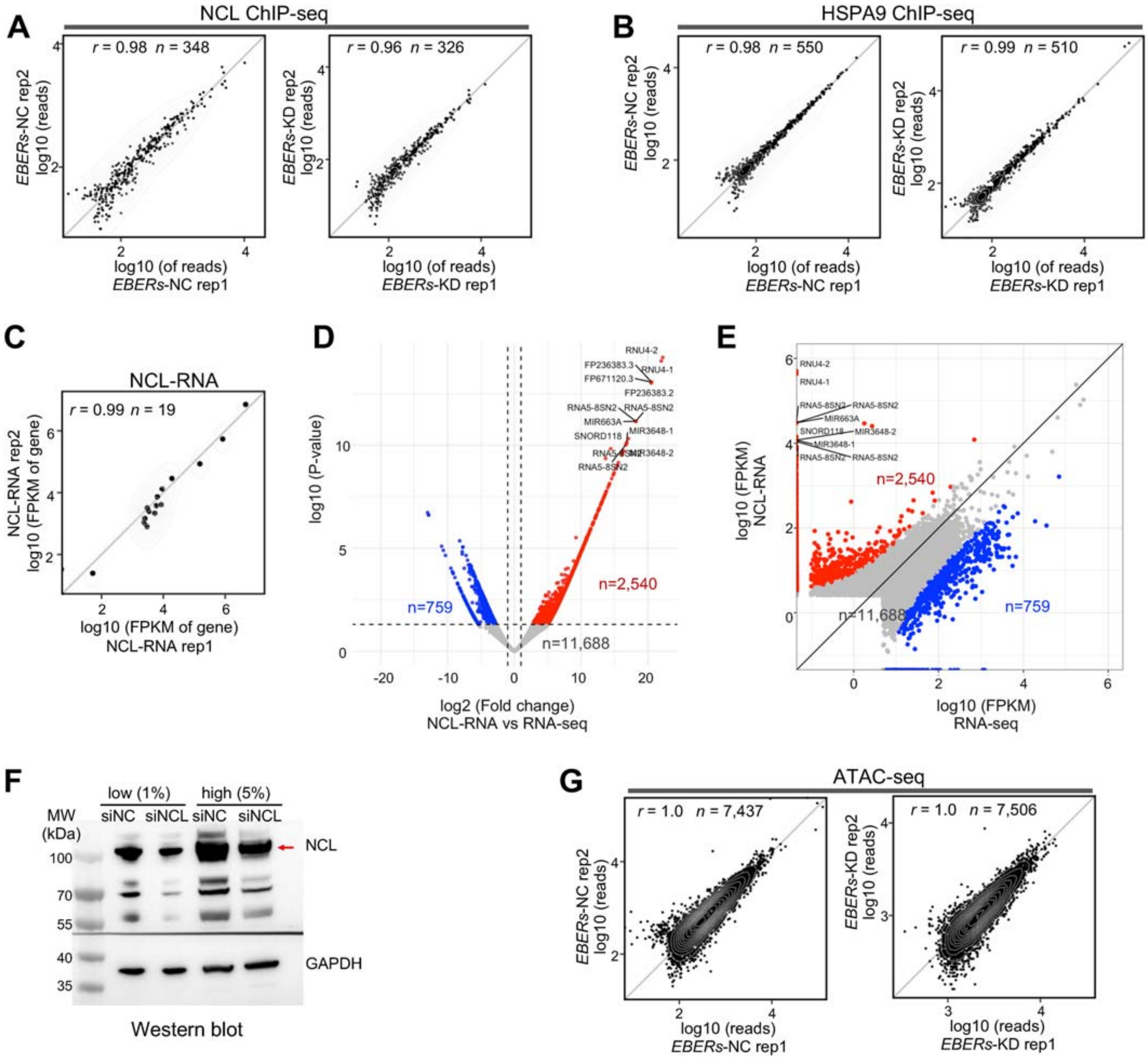

Figure EV10. Reproducibility analyses.

(**A**–**C**) Scatter plots demonstrating the reproducibility of NCL ChIP-seq (**A**), HSPA9 ChIP-seq (**B**), and NCL-interacted chromatin RNA assay captured EBV gene expression associated with NCL protein (**C**) in *EBERs*-NC and *EBERs*-KD. (**D**) Volcano plot illustrating the enrichment of NCL-bound RNAs identified by the NCL-RNA assay compared to RNA-seq. The x-axis represents the log2 fold change (NCL-RNA vs. RNA-seq), while the y axis represents the -log10 P-value from the likelihood ratio test. RNAs enriched in the NCL-RNA data are shown on the right side of the plot, with significantly enriched RNAs highlighted as red points above the horizontal dashed line ($P < 0.05$). The bracket marks the region containing NCL-enriched RNAs. (**E**) Scatter plot showing the gene expression levels (FPKM) of NCL-RNA and RNA-seq, corresponding to (**D**). The scatter plot supplements the volcano plot by providing the original expression levels, which are not visible in the volcano plot. *n* represents the number of genes. (**F**) Western blot showing the knockdown efficiency of NCL using siRNA, with GAPDH as the control. (**G**) Scatter plots illustrating the reproducibility of ATAC-seq in *EBERs*-NC and *EBERs*-KD. “Rep” denotes replicate.

 © The Author(s)
